# Assessing the Utility of Low-Cost Particulate Matter Sensors over a 12-Week Period in the Cuyama Valley of California

**DOI:** 10.3390/s17081805

**Published:** 2017-08-05

**Authors:** Anondo Mukherjee, Levi G. Stanton, Ashley R. Graham, Paul T. Roberts

**Affiliations:** 1 Sonoma Technology Inc., 1450 N. McDowell Blvd., Suite 200, Petaluma, CA 94954, USA; lstanton@sonomatech.com (L.G.S.); paul@sonomatech.com (P.T.R.); 2 Department of Atmospheric and Oceanic Sciences, University of Colorado Boulder, Boulder, CO 80309, USA

**Keywords:** low-cost sensors, performance evaluation, air quality monitoring, pollution, particulate matter

## Abstract

The use of low-cost air quality sensors has proliferated among non-profits and citizen scientists, due to their portability, affordability, and ease of use. Researchers are examining the sensors for their potential use in a wide range of applications, including the examination of the spatial and temporal variability of particulate matter (PM). However, few studies have quantified the performance (e.g., accuracy, precision, and reliability) of the sensors under real-world conditions. This study examined the performance of two models of PM sensors, the AirBeam and the Alphasense Optical Particle Counter (OPC-N2), over a 12-week period in the Cuyama Valley of California, where PM concentrations are impacted by wind-blown dust events and regional transport. The sensor measurements were compared with observations from two well-characterized instruments: the GRIMM 11-R optical particle counter, and the Met One beta attenuation monitor (BAM). Both sensor models demonstrated a high degree of collocated precision (R^2^ = 0.8–0.99), and a moderate degree of correlation against the reference instruments (R^2^ = 0.6–0.76). Sensor measurements were influenced by the meteorological environment and the aerosol size distribution. Quantifying the performance of sensors in real-world conditions is a requisite step to ensuring that sensors will be used in ways commensurate with their data quality.

## 1. Introduction

Air pollution poses a significant global health risk in both developing nations and advanced economies. In a recent epidemiology study, Lelieveld et al. [1] estimated that outdoor air pollution contributed to over 3 million premature deaths in 2010 through cardiovascular and respiratory illnesses, with a majority of deaths occurring in Asia. A recent World Health Organization (WHO) study found that 92% of the world’s population lives in environments where particulate matter (PM) concentrations exceed WHO-recommended levels [2]. 

In the United States, the Environmental Protection Agency (EPA) regulates the ambient concentrations of various air pollutants, including PM (expressed in µg∙m^−3^). The impacts of PM on human health depend on particle size, because fine particles can travel deeper through the respiratory system than larger particles [3]. The health risk of PM is also dependent on the chemical composition of the particles and their toxicology [3]. Size ranges of interest for PM include PM of ~1 µm and smaller in aerodynamic diameter (PM_1_, also called submicron particles), PM of 2.5 µm and smaller in aerodynamic diameter (PM_2.5_, also called fine particles), and PM of 10 µm and smaller in aerodynamic diameter (PM_10_), which have become useful standards for measurements of air quality in the United States and globally. PM_10_ and PM_2.5_ are criteria pollutants for which EPA has established national ambient air quality standards (NAAQS) to regulate concentrations and minimize health impacts. The 24 h NAAQS for PM_10_ and PM_2.5_ are currently 150 µg m^−3^ and 35 µg m^−3^, respectively [4]. In order to ensure these standards are met, instruments that collect data for regulatory purposes must be certified as meeting rigorous quality standards. The EPA designates instruments that have met these standards as federal reference methods or federal equivalent methods (FRM, FEM). The beta attenuation monitor (BAM-1020, Met One Instruments Inc., Grant Pass, OR, USA) has been certified as an EPA FEM instrument for PM_2.5_ and PM_10_, provided it is installed, operated, and calibrated according to established procedures.

Air pollution has become a major concern for the public, as air pollution has received increased media attention, and local and national governments continue to publicize their efforts to improve air quality. Governments and other organizations are also broadcasting air quality data in real time, which has increased public awareness. 

The interest and demand for air quality data has led companies to develop low-cost portable air quality sensors. Some of these sensors are marketed directly to consumers who are interested in their personal exposure. The sensors also have a wide range of potential applications, depending on the quality of the measurements. Because the regulatory monitoring network maintained by the EPA is sparse, with a limited spatial density, low-cost sensors have the potential to provide insight into the spatial and temporal variability of pollutants; data from these sensors could inform studies of personal exposure and emission inventories if the quality of the measurement is robust enough to meet the given objectives [5,6]. Air quality researchers are also examining the deployment of sensor networks and the potential value of integrating these sensors into the existing air quality regulatory network [7,8]. However, different interpretations of data require different levels of confidence in data quality. 

The proliferation of measurements from low-cost air sensors presents data interpretation challenges, as few studies have examined the performance (i.e., accuracy, precision, and reliability) of the sensors under real-world conditions [6]. Quantifying the performance of low-cost air quality sensors is an active area of research, as studies examine sensor measurements in a variety of real-world environments and controlled conditions [9,10,11]. Studies have also examined the performance of a network of sensors, and the use of those measurements to quantify pollution hot spots [12,13]. The validity and uncertainty of sensor measurements over a range of different meteorological and aerosol loading environments needs to be quantified. The need to validate sensor measurements against established methods in real-world environments is key, as laboratory experiments do not typically reflect the variability of meteorological and pollution conditions found in the real world [11,14]. This is a requisite step to ensuring that sensors can be deployed for objectives appropriate to confidence in data quality.

**Figure 1:**
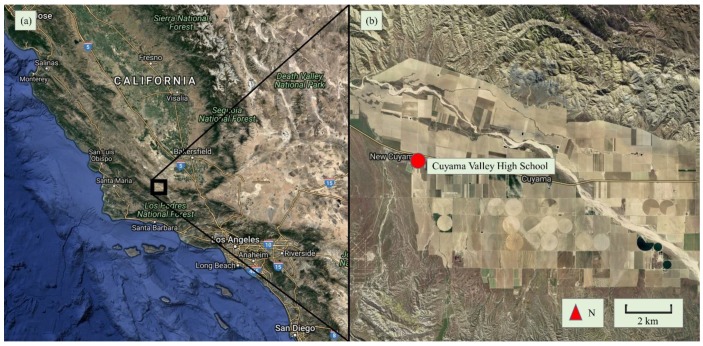
The location of Cuyama Valley in California (**a**); and the location of the field site within Cuyama Valley (**b**).

Santa Barbara County Air Pollution Control District (SBCAPCD) entered into contract with Sonoma Technology, Inc. (STI) to investigate the use of low-cost sensors for monitoring dust by conducting a pilot field study at Cuyama Valley High School in New Cuyama, California. This study evaluated the performance of two low-cost portable PM sensor models: the Optical Particle Counter, OPC-N2 (available for ~450 USD, Alphasense Ltd., Essex, UK) and the AirBeam (available for ~250 USD, HabitatMap Inc., Brooklyn, New York, NY, USA), for detecting particulate matter in the Cuyama Valley of California. Cuyama Valley is a sparsely populated area in southern California, with significant agriculture in the regions of the valley where the topography is flat. Figure 1 shows the general area of the Cuyama Valley and the location of the monitoring site at Cuyama Valley High School in relation to nearby agricultural areas. Major sources of particulate matter include wind-blown dust and regional transport. To examine regional transport patterns, STI modeled transport trajectories for several of the high PM_10_ concentration days identified during this study using the hybrid single-particle Lagrangian integrated trajectory (HYSPLIT) model. Modeled regional transport patterns suggest that, on some days, PM_10_ from the California Central Valley is transported to the New Cuyama area, contributing to the concentrations observed. However, the short-duration high-concentration events reported by the sensors suggest that local transport of dust is also a major contributor to the observed higher PM_10_ concentrations.

The objectives of the study were to determine whether the low-cost PM sensors detect dust events and if so, how well they detect dust events; to determine how precise the sensor measurements are, and whether sensor precision is sufficient either for use in a network to monitor the spatial variability of PM, or to obtain localized data to augment information available from the regional monitoring network; to determine whether the sensors operate continuously and meet data completeness requirements for reliably detecting dust events; and to determine whether the sensors could be used as part of an “early warning system” to inform decisions to reduce exposure to high PM concentrations.

Precision was examined by comparing data from three sensors of the same model. Accuracy was examined by comparing sensor measurements with the FEM BAM, and with the GRIMM 11-R optical particle counter (GRIMM Aerosol Technik GmbH & Co., Ainring, Germany). The impact of meteorology and the variations of the size distribution were also examined. The potential use of these sensors to inform behavior is presented, such as using high temporal resolution to limit exposure.

## 2. Methodology 

STI (Petaluma, CA, USA) deployed six low-cost PM sensor devices and conducted a three-month field study from 14 April 2016, to 6 July 2016, to characterize the performance of the sensors for detecting PM events in the Cuyama Valley environment. Three OPC-N2 and three AirBeam sensors were deployed at Cuyama Valley High School to evaluate sensor performance over a range of conditions. The sensors were collocated with a BAM-1020 measuring PM_10_, a GRIMM 11-R measuring particle sizes, and an R.M. Young 05305V meteorological station measuring wind speed and direction at 1 min temporal resolution. The BAM-1020 was deployed to evaluate the accuracy of the OPC-N2 PM_10_ measurements. Three of each of the two low-cost sensor models were deployed to assess sensor precision and reliability. The GRIMM 11-R was deployed to obtain particle size information and assist with interpretation of sensor performance as a function of particle size. GRIMM 11-R measurements of the particle size distribution were converted to particle mass distribution, including PM_1_, PM_2.5_, and PM_10_.

All of the instruments were collocated within a few feet of one another. Initially, three containers on a small tripod housed one OPC-N2 and one AirBeam sensor each. Each container was equipped with a vent to allow air to be drawn in and a small exhaust fan to blow air out. The meteorological equipment was located on a second tripod. The BAM and GRIMM 11-R were housed nearby in a climate-controlled shelter. Data collected during the study were stored onsite and transmitted in real time to STI’s servers via a cellular modem for archival within a data management system developed for the project. To assess the impacts from sampling orientation on sensor measurements, the sampling orientation for the sensors was varied as shown in Table 1. The reference instruments, BAM and GRIMM 11-R, were oriented with omnidirectional sampling. On 1 June 2016, OPC-N2 A was relocated to a third tripod so that it could sample omni-directionally to assess the impacts of sampling orientation.

Both the AirBeam and the OPC-N2 are optical particle counters (OPCs). An OPC measures the scattered light from a sampled stream of aerosol particles to reconstruct particulate mass concentration [15]. The AirBeam followed an open source development model, so the firmware and the electronic schematics for the instrument are available online. A light-emitting diode (LED) source of visible green light is used to detect particles, and the raw measurement provides particle counts for all particle sizes sampled. In this study, the default conversion algorithm was used to convert these counts (recorded in hundreds of particles per cubic feet, hppcf) to PM_2.5_ mass concentration at a one-minute temporal resolution, which includes assumptions about the particle mass density, refractive index of the particle, and the size distribution. This conversion factor is PM_2.5_ (µg∙m^−3^) = 0.518 + 0.00274 × particle count (hppcf). The AirBeam sensor system also measures temperature and relative humidity [16].

The OPC-N2 uses a laser beam at 658 nm as the light source. The resulting scattered light is focused using an elliptical mirror toward a dual-element photodetector. The firmware of the OPC-N2 is considered proprietary information and includes default settings of 1.5 + 0i for the refractive index of particles and 1.65 g∙cm^−3^ for particle mass density. The refractive index assumption is required because both the intensity and angular distribution of scattered light from the particle are dependent upon it. The OPC-N2 detects particles with diameters within the range of 0.38 µm to 17 µm. Each particle count is classified into one of 16 size bins within this range, resulting in an approximation of the particle size distribution. The firmware calculates values of PM_1_, PM_2.5_, and PM_10_ at a one-minute temporal resolution based on the particle size distribution using the assumed particle mass density value. The OPC-N2 is calibrated by the manufacturer using polystyrene spherical latex particles with a known diameter, refractive index, and density. No correction factor for particle density was applied to the data collected during this study. The assumed particle density of 1.65 g cm^−3^ may be a source of uncertainty for different chemical compositions of PM [17].

PM concentrations were also derived from a GRIMM 11-R OPC. The GRIMM 11-R also measures the particle size distribution through the detection of scattered light. The GRIMM 11-R classifies particle counts into 31 size bins between 0.25 µm and 32 µm at a one-minute resolution. The GRIMM 11-R instrument was designed for the detection of dust particles, which are a major source of PM in the Cuyama Valley environment. Therefore, it is possible that the GRIMM 11-R makes more accurate assumptions of the particle refractive index and in the scattering response of particles than the OPC-N2 and the AirBeam. Concentrations of PM_1_, PM_2.5_, and PM_10_ were computed by converting the particle size distributions into particle volume distributions, using the center of the size bin as the particle diameter for all counts within each respective bin. Then, the total particle volume was computed by summing over the desired size range. A particle density of 1 g∙cm^−3^ was used to convert the measurements to PM concentration values [18].

A BAM-1020 was deployed as a reference instrument to measure PM_10_. The BAM-1020 is a designated EPA FEM for hourly PM_10_ monitoring and is used for over 80% of PM_10_ measurements in the United States at the federal, state, and local levels [19]. Particles larger than 10 μm in diameter are removed by a cyclone, and air is then passed through a chamber that is heated to 20 °C before particles are impacted onto a filter tape that, after a period of collection, is exposed to a source of beta radiation [20]. The degree of absorption of that radiation by particulate matter collected on the filter tape is a sensitive measure of particle mass that is quantified by careful calibration procedures [20,21]. 

Because the instruments use different techniques and have different assumptions in their retrieval of PM values, there are known biases in their measurements depending on the size distribution and chemical composition of the aerosol particles. Because the OPC sensors rely on the detection of scattered light, the measurement of aerosols that are highly absorptive would have a significant low bias. This would be important in environments where the emission of incomplete combustion products leads to a high mass fraction of black carbon in PM. A recent study quantified this impact, showing that OPC-N2-measured values of PM_2.5_ and PM_10_ were a factor of 10 lower for highly absorptive welding fume aerosols than for salt aerosols, compared to the reference measurements by a scanning mobility particle sizer (SMPS) and an aerodynamic particle scanner (APS) [22]. This bias is not likely to be significant in an environment such as Cuyama Valley, where non-absorbing wind-blown dust is a major source of particulate matter. Another source of bias is the variability of the size distribution of the aerosols. Because the GRIMM 11-R has almost twice the size bin resolution as the OPC-N2, we can expect greater accuracy due to the variability in the size resolution. Further, the OPC-N2 would have greater accuracy than the AirBeam due to this bias, because the AirBeam converts particle counts to PM_2.5_ from one size bin of measurements while assuming a predetermined particle size distribution. GRIMM 11-R measurements are used to quantify how the varying particle size distribution can impact sensor measurements of PM.

## 3. Results and Discussion

### 3.1. Cuyama Aerosol Environment

**Figure 2:**
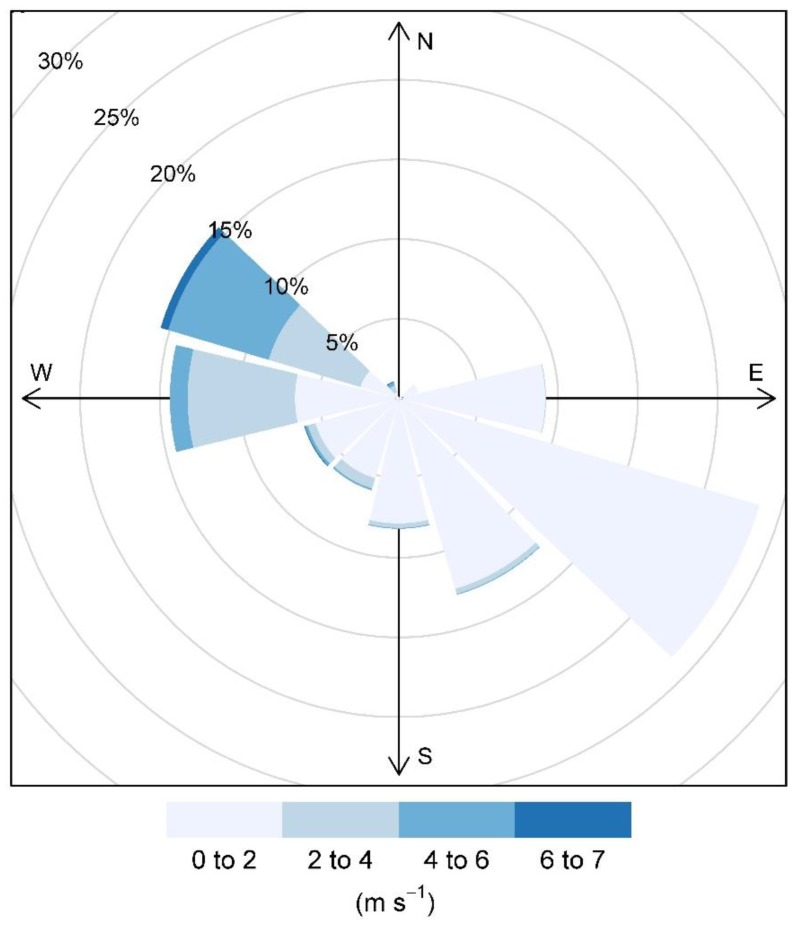
Wind rose for the full study period: 14 April 2016 to 6 July 2016.

**Figure 3:**
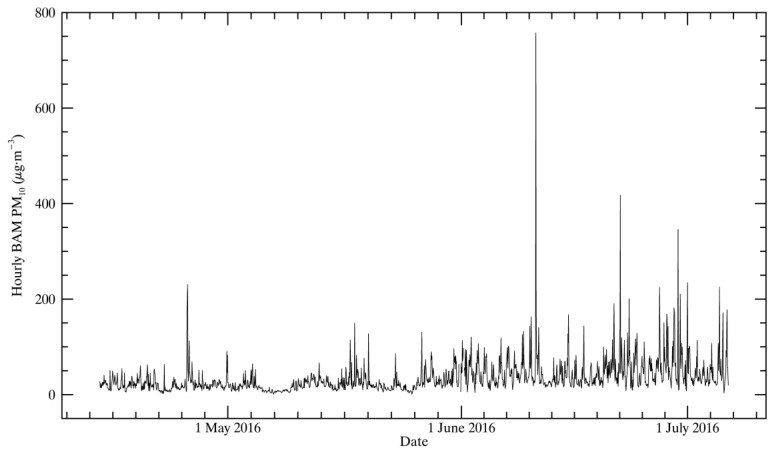
Hourly PM_10_ concentrations measured by the beta attenuation monitor (BAM)-1020 from 14 April 2016 to 6 July 2016.

The wind rose of the full study period in Figure 2 shows low wind speeds (less than 2 m/s) from the southeast or low-to-moderate winds from the northwest the vast majority of the time. Figure 3 shows the hourly PM_10_ concentrations measured by the BAM. Daily average BAM PM_10_ measurements exceeded the threshold of 50 µg∙m^−3^ on 18 out of 84 days (21% of the time). These high PM events, which, for hourly readings, sometimes exceeded 150 µg∙m^−3^, were typically short-term events during low wind speed conditions when winds were from the southeast. Examination of the back-trajectories during high PM periods using the HYSPLIT model showed that some high PM periods correlated with transport from the California Central Valley. This indicates that both wind-blown dust and regional transport contribute to high PM concentrations. High PM events also tend to happen at night or during the early morning in connection with local meteorology and transport patterns.

### 3.2. Precision

The precision of the instruments was evaluated by computing the linear regression and correlation of two of the same instruments. The results, summarized in Table 2, are derived from hourly measurements of PM_2.5_ from the AirBeam and PM_10_ from the OPC-N2.

**Figure 4:**
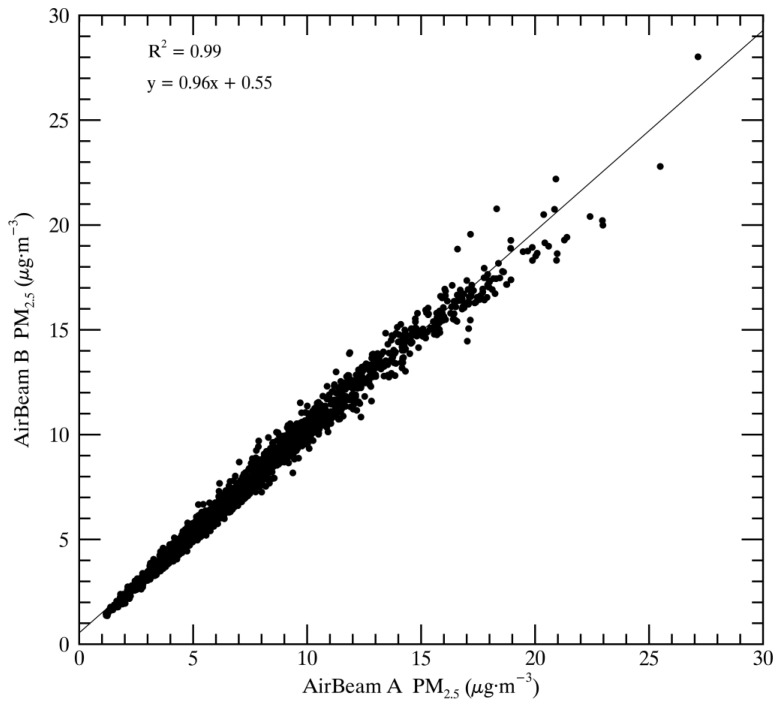
Correlation and linear regression of AirBeam A and AirBeam B PM_2.5_ measurements.

The AirBeams demonstrate a very high precision consistently throughout the study period, over a range of different meteorological conditions and over a range of different aerosol property conditions (chemical composition and size distribution). The close to one-to-one linear regression of AirBeam A vs. AirBeam B shown in Figure 4 is partially due to the fact that they are sampling in the same direction (north) throughout the study. Although AirBeam C is oriented toward the south, we still see a very high precision correlation and linear regression relationship. 

The correlation among OPC-N2s is lower than among the AirBeams. Because OPC-N2 PM_10_ is measuring over a large size range, the natural variability of larger aerosol particles contributes to a lower correlation of OPC-N2 results. It is more challenging to measure a larger range of aerosol particles sizes with high precision.

The OPC-N2 instruments also demonstrate high precision, by correlation. The relationship between the magnitudes of measured PM_10_ also show good agreement, with major differences due to changes in sampling orientation. The large particles, in combination with the sampling orientation differences, are likely two major factors contributing to the range of linear regressions. The one-to-one correlation for OPC-N2 A and B is the highest for the 14 April to 1 June period, the only period when two OPC-N2 instruments were sampling from the same direction (north).

### 3.3. Accuracy Comparison to BAM

The accuracy of the instruments was evaluated by computing the linear regression and correlation of each instrument with the BAM. Table 3 summarizes the results using hourly values, derived from BAM PM_10_, AirBeam PM_2.5_, and OPC-N2 PM_10_.

**Figure 5:**
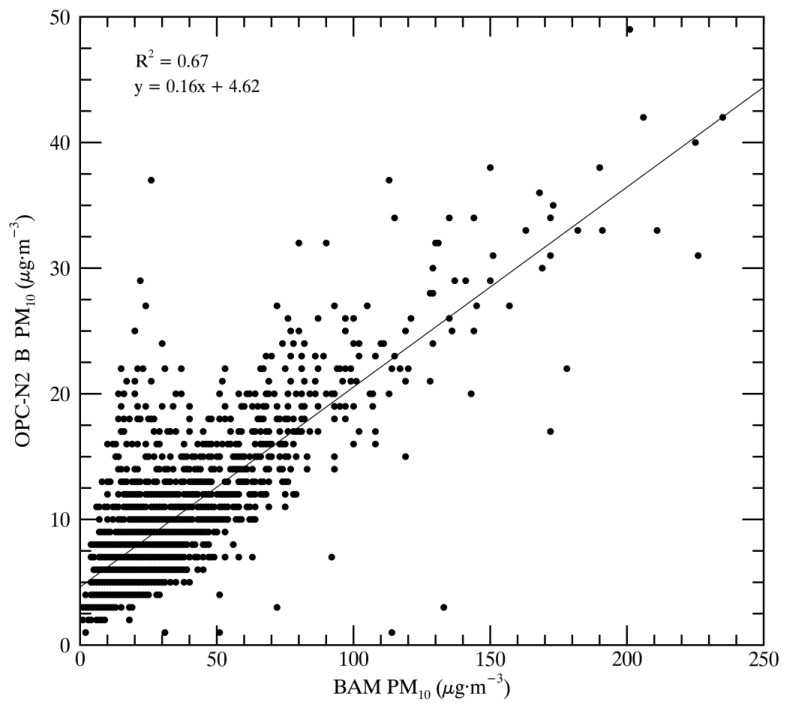
Correlation and regression of OPC-N2 B and BAM-measured PM_10_.

The AirBeams show a lower correlation and associated linear regression against the BAM than the OPC-N2 instruments, partially because the AirBeams are only measuring the PM_2.5_ fraction of PM_10_. The OPC-N2 instruments show higher correlations with the BAM instruments. Figure 5 shows the comparison between BAM and OPC-N2 B-measured PM_10_ throughout the study period. The major factors contributing to the range of linear regressions and the correlation factors are the sampling orientation, the drift of the OPC-N2 instruments (discussed later), and assumptions built into the retrieval algorithm of the OPC-N2. It may be that a bias in the way the OPC-N2 instruments are sampling the size distribution contributes as well. These factors contribute to the OPC-N2 instruments reporting a small fraction of the BAM-measured PM_10_ (approximately 20%).

### 3.4. Sampling Orientation

Sampling orientation is a major contributor to the OPC-N2- and AirBeam-derived correlations and linear regression shown above. Consider the OPC-N2 results shown in Table 2; when OPC-N2 A and B are sampling in the same orientation (14 April to 1 June), the linear regression gives a one-to-one relationship. After OPC-N2 A shifts to an omnidirectional sampling (1 June to 7 July), the relationship changes, with OPC-N2 B reporting 66% of what OPC-N2 A reports. This demonstrates that directional sampling can lead to an underestimation of measured PM_10_. The sampling orientation also contributes to the BAM-derived accuracy relationships. Because the BAM also samples omni-directionally, the change of sampling from north to omni-directional for OPC-N2 A on 1 June increases the coefficient of determination, R^2^, correlation from 0.53 to 0.81. Therefore, it is likely that omni-directional sampling greatly improves the accuracy of the OPC-N2 instrument. These effects are also dependent on the size distribution of the aerosols and the wind direction and wind speed of the meteorological environment.

The correlations and linear regressions of GRIMM 11-R-derived values of PM_10_, PM_2.5_ and PM_1_ versus the BAM and sensor measurements are shown in Table 4. Because the GRIMM 11-R shows a high degree of correlation and accuracy compared to BAM measurements, we can use GRIMM 11-R-derived PM values to evaluate the size dependence of OPC N-2 observations, and compare the relative accuracy of the AirBeam and the OPC-N2 for PM_2.5_ observations. This comparison shows that, while the OPC-N2 reports a fraction of PM_10_, it has a fairly high correlation compared to the GRIMM 11-R. By contrast, the correlation of OPC-N2-measured PM_2.5_ and PM_1_ compared to the GRIMM 11-R is significantly lower, partially because of the downward drift of OPC-N2 values (discussed later). The AirBeam demonstrates a higher accuracy for PM_2.5_ measurements over the course of this study, both in terms of its linear regression and correlation compared to GRIMM 11-R values, in spite of having a simpler measurement technique that does not account for variations in size distribution.

### 3.5. Size Distribution

**Figure 6:**
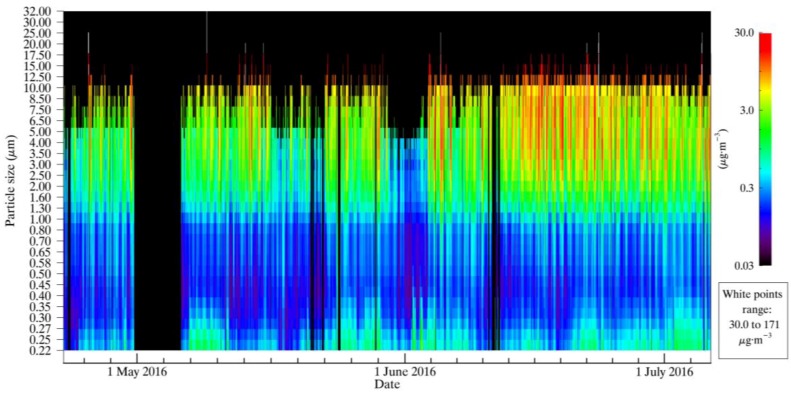
GRIMM 11-R-derived mass distribution over the course of the study.

As the linear regressions from Table 3 and Table 4 show, the OPC-N2 reported a fraction of the PM_10_ values compared to both the BAM and GRIMM 11-R-derived measurements. GRIMM 11-R measurements of the size distribution provide an opportunity to examine the cause of this bias. Figure 6 shows the GRIMM 11-R-derived mass distribution of the aerosol particles, which includes the relative contribution to PM_10_, PM_2.5_ and PM_1_ from each of the size bins measured. Toward the later part of the study period, the GRIMM 11-R-derived mass distribution shows a shift to greater contributions from larger particles. This shows that the precision and accuracy relationships derived previously are valid over a range of different aerosol size distributions.

Given these conditions, it is likely that the OPC-N2 sensors were biased low in their measurement and assessment of the contribution of particles between the 2.5 µm and 10 µm size range to PM_10_. Figure 6 shows that the contribution of these particles to PM_10_ is significant, and the majority of measurements made by OPC-N2 sensors showed PM_10_ less than 50 µg∙m^−3^. One contribution to this bias is the greater size bin resolution of the GRIMM 11-R instrument, which allows for more accurate particle sizing, and thus a more accurate assessment of the contribution of those particles to PM_10_.

**Figure 7:**
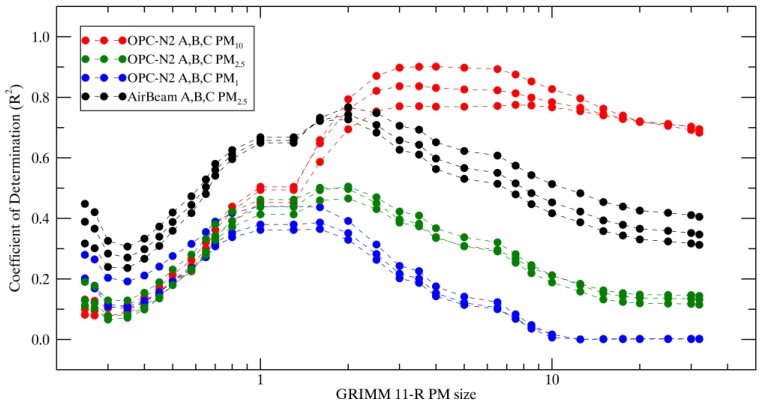
Coefficient of determination (R^2^) of OPC-N2 PM_1_, PM_2.5_, PM_10_, AirBeam PM_2.5_ and GRIMM 11-R-derived PM over a range of size ranges (PM from zero to size).

In order to assess the accuracy of the sensors with respect to particle size, GRIMM 11-R PM concentrations were computed over a range of sizes. For each GRIMM 11-R size bin, the PM concentration up to and including the given size bin was computed (PM_0.25_, PM_0.27_, …, PM_30_, PM_32_, shown in the x axis of Figure 7). The R^2^ between each GRIMM 11-R PM concentration versus OPC-N2 PM_1_, PM_2.5_, PM_10_, and AirBeam PM_2.5_ concentrations are presented in Figure 7. This analysis presents a way of examining the size cut-off of the instruments, and the accuracy of the instruments in sampling the varying size distribution over the course of the study. While the OPC-N2 PM_10_ shows the greatest absolute correlation at the desired size range, the highest correlations are with GRIMM 11-R PM_3_ and GRIMM 11-R PM_6.5_ and the range between them. The lower correlations between the 6.5 µm and 10 µm size ranges indicate that the OPC-N2 was less accurate at sampling PM greater than 6.5 µm, which contributes to the low fraction of PM_10_ that the OPC-N2 observes relative to the GRIMM 11-R and the BAM (shown in Table 3 and Table 4). While absolute OPC-N2 PM_1_ and PM_2.5_ correlations are low compared to GRIMM 11-R PM ranges, they demonstrate accurate cut-off sizing, with correlations peaking at or near the corresponding size bin at 1 µm for PM_1_ and 2 µm for PM_2.5_. The AirBeam also shows an accurate size representation of PM_2.5_ compared to the GRIMM11-R, with correlations peaking at 2 µm.

### 3.6. Meteorology and Size Distribution Influence

**Figure 8:**
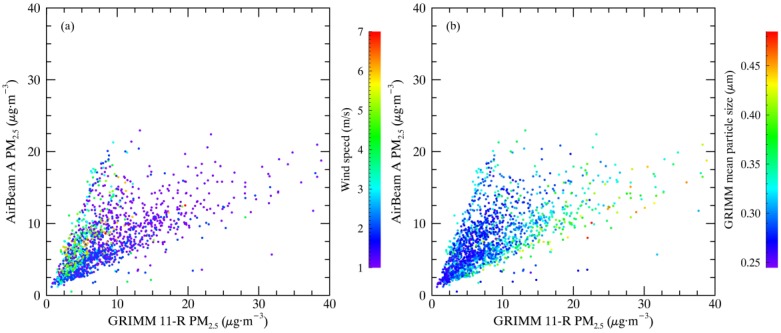
Comparison of GRIMM 11-R- and AirBeam-derived PM_2.5_. Color coding shows wind direction (**a**) and GRIMM 11-R average particle size (**b**).

Figure 8 shows the AirBeam-measured PM_2.5_ and the GRIMM 11-R-derived PM_2.5_. The relationship between AirBeam and GRIMM 11-R-measured PM_2.5_ is a linear response, with a range of values depending on the meteorology and variability of aerosol characteristics, including aerosol chemical composition and size distribution variability. In the right panel of Figure 8, color coding shows the average GRIMM particle size for the full range of GRIMM 11-R size bins. Large average particle sizes lead to lower AirBeam PM_2.5_ measurements relative to the GRIMM 11-R. This reflects the AirBeam measurement technique of detecting total particle scattering—with an assumed size distribution, the AirBeam has the possibility of underestimating or overestimating total PM_2.5_ mass concentration. Figure 8 provides evidence that, for size distributions with larger particles, AirBeams are underestimating PM_2.5_, and, conversely, AirBeams may be overestimating PM_2.5_ for size distributions with smaller particles. However, this one mechanism may not be the dominant driver of the range of values observed, as the variability of the size distribution is interdependent with meteorological conditions, chemical composition variability, and other mechanisms such as hygroscopic aerosol growth. In the left panel of Figure 8, the same AirBeam and GRIMM 11-R PM_2.5_ values are color coded to show wind speed. For lower wind speeds, a trend toward lower AirBeam values is observed, relative to the GRIMM 11-R. Other meteorological variables such as relative humidity may also impact sensor accuracy. Figure 8 shows that both meteorological conditions and aerosol conditions can influence AirBeam measurements, and should be accounted for when sensor measurements are calibrated with established standards.

### 3.7. Data Recovery

Table 5 summarizes data recovery from 14 April 2016, through 6 July 2016. Periods of data loss that occurred during system installation (prior to 14 April 2016) are not included in the number of possible samples since data loss during those periods was not related to the performance of the sensor technology. Data completeness of 75% of 1 min samples was required for each 1 h sample. Data completeness was high (greater than 88%) on both 1 min and 1 h average time frames for all sensors. Several periods of missing OPC-N2 data during the first half of the study were caused by communication issues related to the data logger rather than the OPC-N2 sensor itself. Neglecting data loss related to the data logger issue, data recovery for the OPC-N2 sensors was over 99% for 1-min data and approximately 100% for hourly data.

### 3.8. Drift of OPC-N2 Sensor

**Figure 9:**
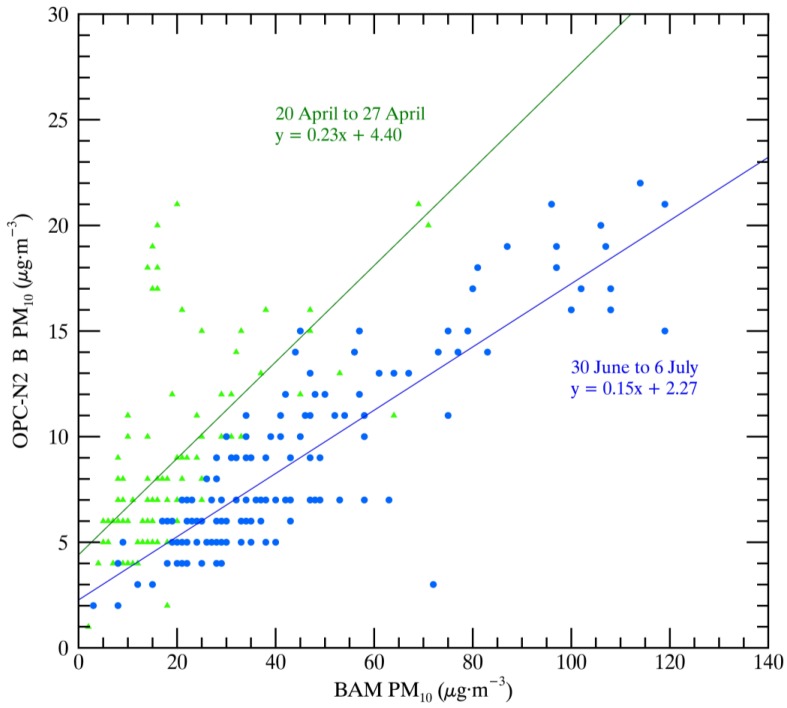
Linear regression of OPC-N2 B versus BAM PM_10_ for the first (green) and last (blue) week of the study.

All three OPC-N2 instruments demonstrated a gradual drift leading to lower PM measurements over the course of the study. Figure 9 shows that over the course of 12 weeks, the relationship between OPC-N2 B and BAM PM_10_ changes, with OPC-N2 B reporting 65% of its initial PM values toward the end of the study.

The drift was quantified in a number of ways: the OPC-N2 PM_10_ values were compared against the BAM PM measurements and the GRIMM 11-R-derived PM_10_. OPC-N2 PM_2.5_ values were compared against GRIMM 11-R and AirBeam PM_2.5_ values. In all cases, a consistent trend exists for the OPC-N2 measurements: a downward drift of the PM values over the course of the 12 weeks relative to all other instruments. The AirBeam instruments do not demonstrate a drift in reported PM values, which is confirmed by comparing AirBeam and GRIMM-derived PM_2.5_.

One possible cause of this downward drift is the buildup of dust on the fan, which would impact the flow rate through the sensor. This may be an acute challenge in the Cuyama Valley environment, because of the long-term presence of dust events with high particulate concentrations and large dust particles. The buildup of dust in the fan would lower the sampling efficiency of the OPC-N2, leading to a smaller fraction of aerosols sampled over time, which would be consistent with observed OPC-N2 performance in this study. Measuring the sample flow rate or routinely conducting maintenance of the OPC-N2 flow system may help to alleviate this effect for future studies in dust-prone environments.

### 3.9. Early Detection 

**Figure 10:**
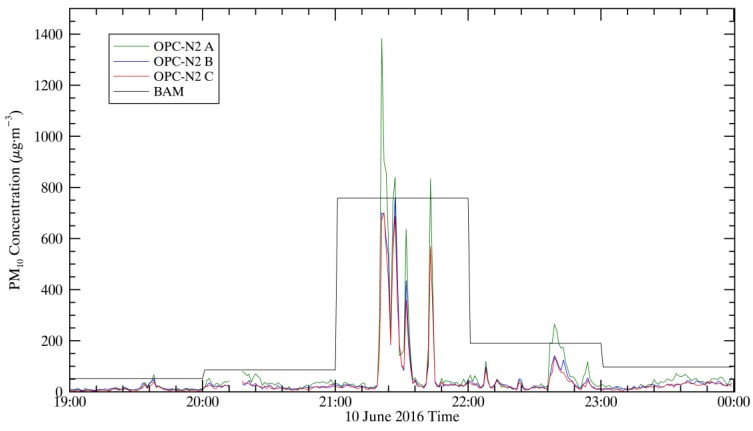
Hourly PM_10_ concentrations measured by the BAM and one-minute PM_10_ concentrations measured by the three OPC-N2 sensors on 10 June 2016. The BAM measurements at 21:00 would have been available at 22:00.

The one-minute resolution of the small sensors gives them the potential to be used as an early detection system for short term events. The Cuyama Valley aerosol environment has significant minute-to-minute variability. Figure 10 demonstrates the ability of the OPC-N2 measurements to detect high aerosol loading events before the BAM has completed its hourly measurement. Because the BAM samples the first 52 min of the hour, for a random distribution of short-term aerosol events, the OPC-N2 instruments would have an average early detection time of 34 min. Because the BAM is not measuring PM during the last 8 min of the hour, the OPC-N2 instruments has a 13% chance of reporting a short-term aerosol event that the BAM fails to measure completely, as was observed in the course of the study.

## 4. Conclusions

Having quantified the performance of the sensors, we present recommendations on the potential utility of the measurements and their deployment in an environment like Cuyama Valley. In Cuyama Valley, there are no air quality monitoring stations in the existing EPA infrastructure; the nearest monitor is in Maricopa, approximately 20 km to the northeast. Cuyama Valley is a unique environment surrounded by mountains, and local air quality measurements could provide valuable information where none exists. While the sensors reported significantly low measurements relative to the GRIMM 11-R and BAM (Table 3 and Table 4), by a factor of 2–4, they could still be utilized as a qualitative measure of high PM events. All sensors and instruments showed coherence in reporting short-term PM events. Furthermore, if the sensors were routinely calibrated relative to a reference monitor, the corrected measurements would be more quantitative, although this calibration may need to be carried out periodically to compensate for the possible influence of drift.

The low-cost sensors demonstrate a robust quality of performance by certain measures, such as high precision and reliability. The OPC-N2 and the AirBeam showed high precision over a range of conditions. The high precision of the sensors (for each sensor model and PM variable) implies that a set of sensors can be utilized to give a consistent, intercomparable measurement which is necessary for being deployed as a network. Sampling orientation was demonstrated to influence the accuracy of the measurements, which has implications for their use as personal sensors or instruments to be integrated into networks. Omnidirectional sampling would improve the consistency of PM measurements.

The accuracy of the OPC-N2 is limited by its drift, which may be due to dust impacting the sampling flow rate. In spite of this drawback, the OPC-N2 demonstrates reasonable correlation against the FEM standard BAM. The low fraction of PM_10_ measured by the OPC-N2 relative to the BAM and GRIMM 11-R is likely due to a combination of drift, sampling of the size distribution, or an internal calibration to a different particle chemical composition/size distribution. The AirBeams demonstrated a higher degree of accuracy for measuring PM_2.5_ compared to GRIMM 11-R-derived PM_2.5_ (compared to the OPC-N2) in the Cuyama Valley environment.

The one-minute time resolution of the sensors relative to the FEM monitors is an advantage. This could be used as an early warning detection system of PM events in Cuyama and in urban environments. This is a unique benefit in the environment of Cuyama Valley, where some high PM events are of very short duration, whereas the BAM reports hourly PM measurements, using the first 52 min of sampling.

The sensors have demonstrated that they are useful for the assessment of short-term changes in the aerosol environment. Further examination of these emerging technologies is necessary, and sensors may be most useful as a supplement to the existing regulatory network. Incorporating collocation with established instruments in the field is encouraged for future studies.

## Figures and Tables

**Table 1:** Instrument sampling orientation.

Instrument	Sampling Orientation
BAM-1020	Omnidirectional
GRIMM 11-R	Omnidirectional
OPC-N2 A	North/Omnidirectional *
OPC-N2 B	North
OPC-N2 C	South
AirBeam A	North
AirBeam B	North
AirBeam C	South

* Between 4 April 2016, and 1 June 2016, OPC-N2 A sampled from the north. On 1 June 2016, OPC-N2 A was relocated to a new tripod, where it sampled omnidirectionally until 6 July 2016.

**Table 2:** Correlation and linear regression of sensor measurements.

X Axis Instrument	Y Axis Instrument	R^2^	Linear Regression	Number of Measurements	Time Period
AirBeam A	AirBeam B	0.99	y = 0.96x + 0.55	1995	full
AirBeam A	AirBeam C	0.98	y = 0.85x – 0.28	1995	full
AirBeam B	AirBeam C	0.95	y = 0.87x – 0.62	1998	full
OPC-N2 A	OPC-N2 B	0.84	y = 0.71x + 3.54	1647	full
OPC-N2 A	OPC-N2 B	0.81	y = 1.00x + 1.95	842	14 April–1 June
OPC-N2 A	OPC-N2 B	0.91	y = 0.66x + 3.45	805	1 June–7 July
OPC-N2 A	OPC-N2 C	0.85	y = 0.57x + 1.72	1629	full
OPC-N2 B	OPC-N2 C	0.79	y = 0.78x − 0.82	1725	full

**Table 3:** Correlation and linear regression of sensor versus BAM PM measurements.

X Axis Instrument	Y Axis Instrument	R^2^	Linear Regression	Number of Measurements	Time Period
BAM	AirBeam A	0.25	y = 0.06x + 5.52	1995	full
BAM	AirBeam B	0.21	y = 0.05x + 5.98	1997	full
BAM	AirBeam C	0.33	y = 0.06x + 4.15	1998	full
BAM	OPC-N2 A	0.76	y = 0.22x + 1.76	1764	full
BAM	OPC-N2 A	0.53	y = 0.21x + 2.71	939	14 April–1 June
BAM	OPC-N2 A	0.81	y = 0.23x + 0.32	825	1 June–7 July
BAM	OPC-N2 B	0.67	y = 0.16x + 4.62	1799	full
BAM	OPC-N2 C	0.61	y = 0.13x + 2.47	1776	full

**Table 4:** Correlation and linear regression of GRIMM 11-R versus sensor and BAM PM measurements.

X Axis Instrument	Y Axis Instrument	Pollutant	R^2^	Linear Regression	Number of Measurements
GRIMM 11-R	BAM	PM_10_	0.91	y = 0.86x + 6.52	1753
GRIMM 11-R	OPC-N2 A	PM_10_	0.84	y = 0.20x + 2.83	1526
GRIMM 11-R	OPC-N2 B	PM_10_	0.81	y = 0.14x + 5.36	1626
GRIMM 11-R	OPC-N2 C	PM_10_	0.81	y = 0.12x + 2.84	1594
GRIMM 11-R	OPC-N2 A	PM_2.5_	0.43	y = 0.15x + 1.92	1521
GRIMM 11-R	OPC-N2 B	PM_2.5_	0.41	y = 0.16x + 3.51	1625
GRIMM 11-R	OPC-N2 C	PM_2.5_	0.40	y = 0.13x + 1.99	1590
GRIMM 11-R	OPC-N2 A	PM_1_	0.39	y = 0.28x + 0.29	1417
GRIMM 11-R	OPC-N2 B	PM_1_	0.45	y = 0.41x + 2.08	1623
GRIMM 11-R	OPC-N2 C	PM_1_	0.38	y = 0.28x + 0.97	1475
GRIMM 11-R	AirBeam A	PM_2.5_	0.66	y = 0.40x + 4.33	1753
GRIMM 11-R	AirBeam B	PM_2.5_	0.62	y = 0.36x + 4.91	1755
GRIMM 11-R	AirBeam C	PM_2.5_	0.71	y = 0.37x + 3.13	1755

**Table 5:** Data recovery for the sensors from 14 April 2016, through 6 July 2016.

Sensor	Number of Possible Samples	Number of Samples Recovered	% Recovery 1-min	% Recovery 1-h
OPC-N2 A	120,181	105,934 *	88.1	88.7
OPC-N2 B	120,181	107,613 *	89.5	95.4
OPC-N2 C	120,181	106,204 *	88.3	92.4
AirBeam A	120,181	119,548	99.5	100.0
AirBeam B	120,181	119,689	99.6	100.0
AirBeam C	120,181	119,689	99.6	100.0

* Data recovery for the OPC-N2 was lower during the first half of the study due to an issue related to communication with the data logger.
